# Mitogen-Activated Protein (MAP) Kinase Scaffolding Proteins: A Recount

**DOI:** 10.3390/ijms14034854

**Published:** 2013-03-01

**Authors:** Melanie Meister, Ana Tomasovic, Antje Banning, Ritva Tikkanen

**Affiliations:** 1 Institute of Biochemistry, Medical Faculty, University of Giessen, Friedrichstrasse 24, 35392 Giessen, Germany; E-Mails: melanie.meister@biochemie.med.uni-giessen.de (M.M.); antje.banning@biochemie.med.uni-giessen.de (A.B.); 2 Department of Molecular Hematology, University of Frankfurt, Medical School, Theodor-Stern-Kai 7, 60590 Frankfurt am Main, Germany; E-Mail: tomasovic@med.uni-frankfurt.de

**Keywords:** mitogen-activated protein kinase, signal transduction, growth factor, receptor tyrosine kinase, scaffolding proteins, cancer

## Abstract

The mitogen-activated protein kinase (MAPK) pathway is the canonical signaling pathway for many receptor tyrosine kinases, such as the Epidermal Growth Factor Receptor. Downstream of the receptors, this pathway involves the activation of a kinase cascade that culminates in a transcriptional response and affects processes, such as cell migration and adhesion. In addition, the strength and duration of the upstream signal also influence the mode of the cellular response that is switched on. Thus, the same components can in principle coordinate opposite responses, such as proliferation and differentiation. In recent years, it has become evident that MAPK signaling is regulated and fine-tuned by proteins that can bind to several MAPK signaling proteins simultaneously and, thereby, affect their function. These so-called MAPK scaffolding proteins are, thus, important coordinators of the signaling response in cells. In this review, we summarize the recent advances in the research on MAPK/extracellular signal-regulated kinase (ERK) pathway scaffolders. We will not only review the well-known members of the family, such as kinase suppressor of Ras (KSR), but also put a special focus on the function of the recently identified or less studied scaffolders, such as fibroblast growth factor receptor substrate 2, flotillin-1 and mitogen-activated protein kinase organizer 1.

## 1. MAP Kinase Signaling Cascade

### 1.1. Components of the MAPK/ERK Signaling Cascade

The process of signal transduction through receptor tyrosine kinases (RTKs) starts with an extracellular stimulus that activates its designated receptor and, thereby, initiates downstream signaling mechanisms. This can be established by a sequential phosphorylation of a three-tier kinase cascade, which conveys the signal to the nucleus, where a cellular response can be transcriptionally mounted. Protein kinases catalyze the transfer of a γ-phosphoryl group from ATP to a hydroxyl-group of a serine, threonine or tyrosine residue in the substrate protein. They are named after their substrate specificity, *i.e.*, protein serine/threonine kinases, including dual specificity kinases, protein tyrosine kinases and tyrosine kinase-like proteins [1]. Since phosphorylation is a reversible process, phosphatases provide the counterpart to kinases [2].

**Figure 1:**
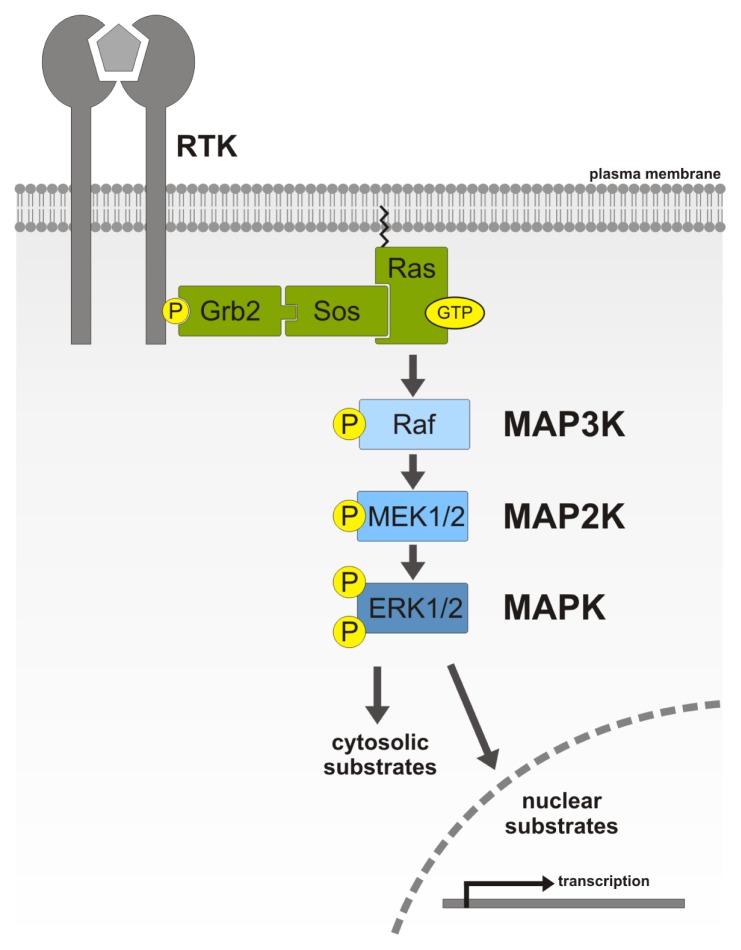
Schematic representation of the extracellular signal-regulated kinase (ERK)/mitogen-activated protein kinase (MAPK) cascade. Activation of receptor tyrosine kinases (RTK) results in recruitment of the adaptor, growth factor receptor-bound protein 2 (Grb2) and the guanine exchange factor (GEF), son of sevenless homologue (Sos), which then interacts with and activates Ras. This results in activation of C-Raf and, thereby, the initiation of sequential phosphorylation steps of the MAPK cascade. Activated ERK can phosphorylate either cytosolic or nuclear substrates.

A prime example for such a kinase cascade is the mitogen-activated protein kinase (MAPK) pathway, an evolutionarily highly conserved signaling mode that controls fundamental cellular processes, such as proliferation, cellular survival and differentiation. A deregulation of one component in this cascade can result in aberrant signaling events associated with a pathological outcome, e.g., cancerogenesis. Three protein families are central to this pathway, *i.e.*, the extracellular signal-regulated kinase (ERK) family, the p38 kinase family and the c-Jun *N*-terminal kinase (JNK) family. The MAPK cascade itself is shaped by three major constituents that form the so-called “three-tiers”, MAPK kinase kinase (MAP3K), MAPK kinase (MAP2K) and MAPK (Figure 1). In addition, several other regulators, activators and scaffolding proteins can affect this cascade. Downstream of MAPK, both cytosolic and nuclear substrates can be phosphorylated. Located upstream of the MAPK tiers are, e.g., the transmembrane RTKs, integrins and G protein-coupled receptors (GPCR), which, upon activation, initiate the downstream signaling and therewith successively recruit the MAPK components to signalosomes. Ligand binding to monomeric transmembrane RTKs, such as epidermal growth factor receptor (EGFR), induces receptor dimerization [3] and results in transphosphorylation of the receptor molecules [4,5]. The link between the activated, phosphorylated EGFR and the GTPase Ras (p21), which resides immediately upstream of the starting point of the MAPK pathway, is established by two proteins, growth factor receptor-bound protein 2 (Grb2) and son of sevenless homologue (Sos) [6–9]. The adapter protein Grb2 utilizes its Src homology (SH) domains to bind to the phosphorylated tyrosine 1068 of EGFR (SH2 domains), as well as to a poly-proline region in Sos (SH3 domain) [10,11]. Sos is a guanine exchange factor (GEF) for Ras [12–14], whose GTP loading is essential for the Ras-dependent activation of the MAPK pathway, since dominant negative Ras mutants are unable to activate downstream signaling [15]. The GTP-bound, active form of Ras then directly binds to the Ras-binding domain (RBD) of the serine/threonine kinase Raf [16–20] and recruits it to the plasma membrane [21–23]. Inactive Raf is bound to a regulatory protein, 14-3-3, in the cytosol [24], but upon Ras-induced membrane recruitment, Raf is released from 14-3-3 [25].

Three different isoforms of the MAP3K Raf exist, namely C-Raf (also known as Raf-1), A-Raf and B-Raf, which provide the first tier in the MAPK cascade. Although Ras is involved in the canonical Raf activation, it is not essential for MAPK signaling. Some signaling pathways, e.g., protein kinase C (PKC) signaling, which can be activated with phorbol myristyl acetate (PMA), result in Raf activation without the requirement for the preceding activation of Ras [26,27]. Similar to a dominant-negative Ras, blocking Raf activity, e.g., by expressing a dominant negative mutant, results in an impairment of the activation of the MAPK pathway upon growth factor stimulation [28].

Upon its activation, Raf transmits the signal further downstream to MEK1/2 (MAPK/ERK kinases 1 and 2), which are the second tier in the MAPK cascade. MEK1/2 (also known as p45/p46) are activated by Raf-mediated phosphorylation at Ser218 and Ser222 (hMEK1) [29–32]. MEK1 and MEK2 are highly homologous dual specificity kinases, which are capable of phosphorylating both tyrosine and serine/threonine residues [33]. Astoundingly, however, their only known substrates are the MAPK ERK1/2 (p44/p42). MEK1/2 phosphorylate human ERK1 within the Thr202-Xaa-Tyr204 motif [34–36]. Activated ERK1/2 are released from MEK1/2 and either phosphorylate target substrates in the cytosol or translocate to the nucleus [37–39]. In contrast to MEK1/2, ERK1/2 have a plethora of substrates and are, therefore, effectors of many cellular processes, e.g., differentiation and proliferation [40,41].

### 1.2. Function and Regulation of ERK

There are eight isoforms of the MAPK ERK, ERK1-8. In this review, we focus on ERK1 and ERK2, as their function has been extensively characterized and they represent typical components of the canonical MAPK signaling pathway. ERK1/2 are serine/threonine protein kinases [42] that prefer to phosphorylate their substrates at Ser/Thr-Pro motifs [43,44]. Recently, novel ERK1 splice variants that are cell-type specific and less abundant were described [45,46]. Due to their high degree of homology, ERK1 and ERK2 have been suggested to share most of their functions. Stimulation of cells generally results in activation of both ERK1 and ERK2, and it is, thus, under debate whether ERK1 and ERK2 are functionally redundant when expressed in the same cell type or if they can indeed exert different functions [47–52]. Lefloch *et al.* suggested that functional differences between ERK1 and ERK2 would depend on their expression levels [48]. Von Thun *et al.* used a model system for tumor invasiveness to show that depletion of ERK2 impairs invasive migration of the cells. Strikingly, the invasive motility could only be rescued by ERK2, but not by ERK1, again implicating that the two ERK proteins exhibit functional differences [53]. However, the most striking hints for the distinct functions of ERK1 and ERK2 came from the respective knockout mice. While ERK1^−/−^ mice are viable and exhibit relatively minor defects, e.g., in thymocyte differentiation [54], ERK2^−/−^ mice already die at the embryonic stage, E8.5 [55]. This implies that in the ERK2 knockout mice, ERK1 is not able to compensate for the loss of ERK2. However, it has not been clarified if this is due to a lack of redundancy or a missing expression of ERK1 in certain cell types in the ERK2 knockout mice and, thus, failure to compensate for the non-existing ERK2 activity.

The subcellular localization of inactive and active ERK1/2 reflects their broad functional effects. About 10–20 min after the cells are exposed to a growth factor/mitogen, active ERK1/2 translocate to the nucleus, where they induce gene expression and facilitate cell cycle entry. It is still under debate whether ERK translocates to the nucleus as a homodimer or in its monomeric form [37–39,56–58]. The nuclear translocation of ERK is either facilitated by passive diffusion or by an active transport mechanism [59]. The active translocation of ERK to the nucleus requires phosphorylation of two serines within its nuclear translocation signal (NTS), which is mediated by casein kinase 2 (CK2), binding to importin-7 and phosphorylation of nucleoporin 50 (NUP50) [60–63]. Recently, another protein of the nuclear pore complex, TPR (translocated promoter region) was identified as an ERK2 substrate that influences nuclear translocation of ERK2 [64,65]. ERK is involved in several nuclear processes, including transcriptional regulation of gene expression. Transcription factors, such as the Ets-domain containing protein, Elk-1, can be phosphorylated by ERK1/2 [66–69], and the phosphorylated Elk-1 then initiates the transcription of immediate-early response genes (IEG), such as c-Fos [70,71]. Fos itself is able to subsequently activate delayed early genes (DEG), including the dual specificity Thr/Tyr MAPK phosphatase 6 (DUSP6). Together with some other members of the DUSP family, DUSP6 is known to dephosphorylate ERK1/2 with high specificity and, thus, functions as a negative feedback regulator of MAPK signaling [72–74].

ERK1/2 do not only enhance gene transcription, but can also act as transcriptional suppressors by phosphorylating the Ets2 repressor factor (ERF) [75]. Furthermore, ERK1/2 can target and activate several nuclear receptors, e.g., estrogen receptor (ER), a receptor upregulated in breast cancer [76,77], or peroxisome proliferator-activated receptor gamma (PPARγ), a receptor involved in diabetes and obesity [78]. In addition, ERK1/2 regulate chromatin remodeling by activating DNA-binding proteins like poly-ADP-ribose polymerase 1 (PARP-1) [79] and modulate histone modification by phosphorylating mitogen and stress activated protein kinases 1 and 2 (MSK1/2) [80–82]. However, the activation of PARP-1 was shown to be independent of ERK2 kinase activity, as such; yet, it seems to depend on an active, phosphorylated ERK [79]. PARP-1 itself has been discussed to be an anchoring protein that would keep ERK in the nucleus and, thereby, facilitate the interaction of ERK with Elk-1 [79,83].

In addition to the manifold substrates of ERK in the nucleus, it also has cytosolic substrates and localizes to other subcellular compartments, such as endosomes, via the MP1-p14 complex, and mitochondria, via voltage-dependent anion channel 1, VDAC1 [84–88]. In the cytosol, ERK1/2 have several substrates, some of which will be discussed below. For a more detailed discussion of the topic, please see the recent reviews [41,89]. Recently, Asano *et al.* described the actin-binding protein, palladin, as a novel substrate for ERK and suggested an anti-migratory function for ERK [90]. Another cytosolic substrate and a scaffolder of ERK is paxillin, a protein found to associate with focal adhesion kinase (FAK) in focal adhesions upon stimulation of cells with hepatocyte growth factor (HGF) and to enhance cell spreading and adhesion [91]. Amongst the first described cytosolic substrates of ERK is the family of 90 kDa ribosomal S6 kinases (RSK), a group of Ser/Thr kinases. These proteins are downstream effectors of the MAPK cascade and involved in several cellular processes, e.g., cellular proliferation and differentiation [92,93].

The MAPK cascade is a multifaceted pathway that is involved in a plethora of cellular processes. A deregulation of this pathway results in pathologies, for instance, in cancerogenesis. Regarding the substrate specificities within this pathway, ERK and MEK are in great contrast to each other. While ERK1/2 are the only described substrates of MEK1/2 so far, ERK1/2 has a myriad of substrates in the cytosol and the nucleus. Therefore, a constant feedback mediated regulation of this pathway is important. There are two different regulatory mechanisms: a positive feedback mechanism, which results in an increase in ERK signaling, and a negative feedback mechanism by which ERK signaling is limited and returned to a basal level. For example, ERK1/2 is able to phosphorylate MEK1 at Thr292 and Thr386 *in vitro*[94,95]. *In vivo*, ERK1/2 phosphorylates MEK1 as part of a negative feedback regulation during integrin and growth factor signaling [94,96]. Recently, formation of MEK1-MEK2 heterodimers was reported, which was found to be negatively regulated by ERK1/2 by phosphorylation of Thr292 in MEK1, a residue that is not present in MEK2 [97]. Another ERK1/2 mediated negative feedback regulation is the phosphorylation of Sos, which in turn interferes with the interaction of Sos and Grb2 [98]. In general, negative regulators of kinase activities are phosphatases. In particular, dual specificity Thr/Tyr MAPK phosphatases (DUSPs or MKPs) display a negative effect on ERK1/2 activity (for a review on DUSPs, please see [99]). Like ERK1/2, DUSPs can localize to both the cytosol and nucleus. For example, DUSP1 itself is a substrate of ERK1/2. However, phosphorylation stabilizes nuclear DUSP1, which ultimately can lead to a dephosphorylation of ERK1/2 [100,101]. On the other hand, cytosolic ERK1/2 phosphorylate DUSP6 on Ser159 and Ser197 and target it for proteasomal degradation, providing a positive feedback on MAPK signaling [102]. Aside from nuclear regulators of ERK, Sef is an example of a subcellular regulator of ERK, which is associated with membrane ruffles and the Golgi. Sef blocks the nuclear translocation of ERK1/2 by binding it when it is still in complex with MEK1/2 and, thus, inhibits the dissociation of ERK and MEK [103–105].

## 2. MAP Kinase Scaffolders

### 2.1. MAPK Scaffolding Proteins—An Introduction

**Figure 2:**
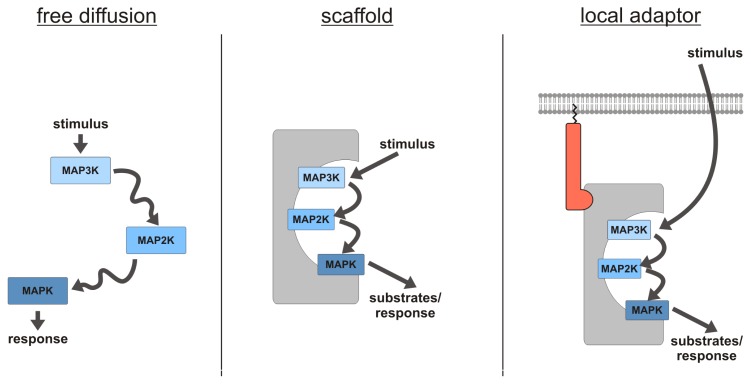
Characteristics of scaffolding proteins. (**left**) The model postulating a free diffusion of signaling proteins is not able to sufficiently explain the efficiency of signaling; (**middle**) scaffolding proteins contain several domains for the binding of their interactors and serve as platforms for efficient signal transduction; (**right**) local adaptors function as specific subcellular anchors for the scaffolding protein and its interactors to cellular subcompartments, such as the plasma membrane or endosomes.

The model postulating a free diffusion of signaling components in the cytosol comes along with several disadvantages (Figure 2). The probability for two consecutive kinases to meet in the cytosol is fairly low, and diffusion based signaling would be extremely inefficient. In addition, the signal needs to be transmitted to the corresponding subcellular region. Therefore, scaffolding proteins support signaling partners in propagating the signal and in directing it to the correct cellular location. Scaffolds are defined as proteins with several domains that bind two or more components of a signaling pathway simultaneously. They bring signaling partners in close proximity to each other, link them in a multi-enzyme complex and facilitate their functional interaction. Within this complex, the kinases are shielded from the deactivating phosphatases, and interference with other signaling cascades is minimized [106,107]. Since not all stimuli result in the same cellular response, different scaffolds anchor the signaling modules, with the assistance of various adapter proteins, to specific subcellular regions and enhance the signaling by providing a platform for the interaction with their respective substrates. Altogether, scaffolds enable a plethora of signaling variations by linking the same core kinases in a different subset of reaction partners, substrates and cellular surroundings.

One main characteristic of scaffolds is combinatorial inhibition, an effect first described for the ERK/MAPK scaffold kinase suppressor of Ras (KSR) [108]. Combinatorial inhibition describes the stoichiometry of a scaffold and its signaling partners [109,110]. If the scaffold concentration is too low, the scaffold-dependent-enhanced binding of the kinase and its substrate is not given and, thus, the signaling is below optimum. On the contrary, if the scaffold is in excess, the kinase and its substrate will each bind to an individual scaffold. Hence, downstream signaling is impaired in the absence of a productive interaction. It is in most cases largely unknown how scaffolds themselves are regulated. In a recent review, Dard and Peter discuss mechanisms and modifications that seem to play a regulatory role during scaffold turnover. These mechanisms include oligomerization, conformational activation, nucleocytoplasmic shuttling and phosphorylation [111].

The first MAPK scaffold described was the yeast Ste5p protein. However, in this review, we will focus on MAPK/ERK scaffolds in mammalian cells, and thus, Ste5p, as well as the scaffolds of the JNK MAPK pathway, will not be discussed. For Ste5p and JNK scaffolds, we refer to some recent reviews on these topics [112–114]. Several scaffolding proteins for the ERK/MAPK cascade have been described, with KSR (kinase suppressor of Ras) and ß-arrestin being probably the best known ones. Lately, some proteins with scaffolding characteristics have been described, such as connector enhancer of KSR 1 and 2 (CNK1/2) and IQ motif containing GTPase-activating protein 1 (IQGAP1). However, these proteins appear to bind only to some of the core kinases of the MAPK pathway and are, thus, considered to be “nested scaffolds” [115]. IQGAP1, a multidomain protein with several protein interaction motifs, was shown to interact with B-Raf, C-Raf, MEK1/2 and ERK1/2 [116–119]. In fact, IQGAP1 appears to be necessary for the growth factor-dependent activation of B-Raf [117] and to promote a MEK1-dependent signaling pathway [118,119]. In the following part, we will summarize the established ERK scaffolding proteins, such as KSR1, MEK-binding partner 1 (MP1) and ß-arrestin, but also shed light on some less studied or recently identified scaffolding proteins, such as mitogen-activated protein kinase organizer 1 (MORG1), fibroblast growth factor receptor substrate 2 (FRS2) and flotillin-1.

### 2.2. Kinase Suppressor of Ras

Initially identified in genetic screens in *Drosophila melanogaster* and *Caenorhabditis elegans* as a modifier of activated Ras, KSR1 was found to be a positive regulator of MAPK signaling and represents one of the best characterized MAPK scaffolders so far [120–123]. In *D. melanogaster*, only a single *ksr* gene is present, which is essential for the viability of the organism. On the other hand, in mammals and *C. elegans*, two proteins, KSR1 and KSR2, are present and display a functional redundancy [122,124,125]. Whereas the loss of KSR is embryonically lethal in *D. melanogaster*, the loss of KSR1 has little effect in *C. elegans*[120–123]. Similarly, KSR1 knockout mice are viable and without any major developmental defects [126,127], suggesting a large degree of functional redundancy of the KSR isoforms. Functional implications for KSR1 were acquired by the fact that these mice display defects in T-cell activation and neuronal signaling [128,129] and a decrease in tumor formation caused by polyomavirus middle T (MT) or by treatment with 12-O-tetradecanoylphorbol-13-acetate (TPA) [127,128]. Furthermore, in the absence of KSR1, the induction of arthritis is impaired [130] and KSR1 knockout mice have enlarged adipocytes, indicating a possible role of KSR1 in adipogenesis [131]. The ksr1^−/−^ mice are modestly glucose intolerant. Although they show a normal response to exogenous insulin, they display a three-fold increase in serum insulin levels in response to glucose challenge [126], demonstrating another important role of KSR1 in insulin-regulated glucose metabolism. Most importantly, in KSR1 knockout mice, the high molecular weight complexes containing KSR, MEK and ERK are lost [128]. All this unravels KSR1 as a molecular scaffold that is not strictly required for, but rather enhances, signaling via the MAPK signaling pathway.

KSR1 is able to bind all three kinases of the MAPK pathway. Whereas it constitutively associates with MEK, it interacts with C-Raf and ERK only upon growth factor stimulation [132,133]. With this, it enables MEK to come in close proximity to the Raf kinase at the plasma membrane, which executes its phosphorylation. The domain organization of KSR proteins is similar to that of the Raf proteins, comprising five conserved regions, termed CA1–CA5. The CA1 domain of *Drosophila* KSR was shown to bind D-Raf (reviewed in [134]), but this region is absent from the KSR proteins of *C. elegans*. CA2 contains a proline rich region, whereas CA3 is a cysteine rich, atypical C1 domain, displaying homology to the one found in Raf proteins. The CA3 domain regulates the cellular localization of KSR and Raf by assisting in the membrane anchorage following growth factor stimulation or Ras activation [135]. Finally, the CA5 domain is closely related to the kinase domain of Raf proteins and seems to be important for constitutive interaction with MEK [108,136–138]. Upon its discovery, KSR1 was considered to be a pseudokinase that lacks catalytic activity, and up to date, the evidence for its catalytic activity is not completely unambiguous [123,135,137,139–142]. The lack of catalytic activity of KSR was suggested to rely on the fact that an invariant Lys residue, which is important for orienting ATP in Raf [143], is exchanged for an Arg in KSR1, and despite its similar character, the Arg seemed not to be able to complement the Lys as a catalytic residue [144]. However, recent studies have provided some experimental evidence for the catalytic activity of both KSR1 and KSR2 [145,146]. A KSR1 mutant that is not capable of binding ATP, but associates with both C-Raf and MEK1, fails to activate MEK1, implicating that KSR1 catalytic activity, or at least its ATP binding capability, is required for MEK1 phosphorylation [146]. Furthermore, recent findings have suggested that recombinantly expressed, purified wild-type KSR1, but not its mutant form, is capable of phosphorylating MEK1 *in vitro*[140]. It has been suggested that Raf kinases are important for the kinase activity of KSR by their virtue of binding to KSR and allosterically stimulating its catalytic activity. Hu *et al.* have recently shown that MEK1 phosphorylation by KSR1/C-Raf heterodimers requires KSR activity and cooperation with C-Raf [146]. Likewise, a kinase-deficient form of B-Raf was capable of stimulating MEK1 phosphorylation by KSR2, implicating that Raf activity may not be a prerequisite for MEK phosphorylation in this complex [145]. Thus, although these data suggest that KSR may be catalytically active, this appears to require some kind of cooperation, and most likely, dimerization with Raf kinases and the molecular details of KSR regulation will surely be an important aspect of future research.

Depending on its phosphorylation state, KSR1 is found to be localized at the plasma membrane or in the cytoplasm. As with Raf, KSR is held in the cytoplasm by the bound 14-3-3 protein, but it translocates from the cytoplasm to the plasma membrane upon treatment with growth factors in a process regulated by Cdc25C-associated kinase 1 (C-TAK1). C-TAK1 is constitutively associated with KSR1 and phosphorylates its Ser392 residue. Since Ser392 is the site that mediates the binding to 14-3-3 proteins, phosphorylation of this residue by C-TAK1 governs cytoplasmic sequestration of KSR1 in unstimulated cells [133]. Upon growth factor stimulus, Ser/Thr protein phosphatase PP2A dephosphorylates KSR1 on the critical Ser392 residue, resulting in displacement of 14-3-3 and release of the inhibition imposed by 14-3-3. Thus, KSR1 is now free to promote MAPK pathway activation [147].

Apart from 14-3-3, another KSR1 inhibitor has been identified. IMP (impedes mitogenic signal propagation) inhibits signal propagation by disrupting KSR1 homo-oligomerization, which is necessary to join Raf with its substrate, since it seems that different complexes of KSR1-MEK, KSR1-C-Raf and KSR1-B-Raf exist in the cells (reviewed in [148]). Thus, IMP acts as a signal threshold regulator and uncouples C-Raf from KSR1 complexes [149].

### 2.3. MEK Partner 1/p14 Complex

As already mentioned, the three tiers of the MAPK signaling cascade are initially activated at the plasma membrane. However, at later stages, the complexes are present in endosomes, which is required to achieve a proper signaling response. It seems that the endocytosis of the activated receptors and their associated signaling complexes is crucial for maximal MAPK activation, and activated Ras, C-Raf, MEK1 and ERK1 can be found on endosomes [138,150–153]. The reasons for the presence of the second MAPK activation phase, which takes place at the so-called signaling endosome, might be multiple. Endocytosis might enable a more controlled spatio-temporal resolution of the signaling cascade. Furthermore, it might also shield the signal during transmission over long distances, whereas slow protein diffusion and rapid protein dephosphorylation might interfere with it.

MEK partner 1 (MP1) is a widely expressed small protein that was originally identified in a yeast-two hybrid screen using MEK1 as a bait and was correspondingly named after its ability to bind to MEK1 [85]. MP1 facilitates a transient binding of MEK1 to ERK1, thereby enhancing the activation of the MAPK signaling cascade, but it does not bind MEK2 or ERK2 [85]. The group of Huber identified the scaffold protein MP1 as a putative interaction partner of p14, which is a highly conserved protein of 14 kDa [86]. In different cell types, p14 was found to be localized at the cytosolic face of the late endosomes/lysosomes [86]. The interaction of MP1 with p14 was found to be important for the localization of the MP1-MAPK scaffold complex to late endosomes/lysosomes during the second sustained phase of MAPK signaling [154]. However, p14 was found to interact with MEK1 and ERK1 only indirectly via MP1, despite the incredibly similar structures of p14 and MP1, exhibiting a five stranded beta sheet flanked on each side by three helices [155].

Depletion of p14 and MP1 led to a pronounced inhibition of the MAPK signaling pathway. Interestingly, reduced ERK activation during the later stages of signaling was observed, while the early phase of ERK activation at the plasma membrane was unaffected. However, overexpression of p14 was not sufficient to inhibit MAPK signaling, pointing to the fact that p14 does not disrupt the formation of signaling complexes in the way a highly overexpressed MP1 or other scaffolds do [154].

Consistent with its role in endosomal localization of MP1, p14 knockdown resulted in a mislocalization of MP1-MAPK scaffold complexes from late endosomes to the cytoplasm. However, p14 does not contain clearly identifiable membrane localization signals, yet it appears to be important for the localization of the complex in the membranes. Later findings have shown that the endosomal localization of MP1/p14 complexes is facilitated by a small adaptor protein, p18, which is associated with lipid rafts by means of myristoylation and palmitoylation [84]. Interestingly, endosomal dynamics and endosomal association of MP1/p14 complexes were inhibited in cells depleted of p18. Mice lacking p18 expression show an early embryonic lethality due to an impairment of the development of the visceral endoderm and the resulting growth arrest. Cell lines established from the p18 knockout embryos display severe abnormalities in the endosomal/lysosomal compartments, associated with peripheral scattering of the normally perinuclear endosomal compartments. Since p18 is required for the endosomal localization of the MAPK scaffolding complex, MP1/p14, these data suggest that endosome-associated MAPK signaling may play a role in the regulation of the biogenesis of endosomes and lysosomes. Highly intriguingly, a mutation in the 3′ untranslated region of the p14 mRNA that results in reduced p14 expression was found to cause a human genetic disease that manifests as a primary immunodeficiency associated with albinism, short stature and defects in B-cell and cytotoxic T-cell function [156]. In fibroblasts lacking p14 expression, ERK phosphorylation was reduced, in line with the function of p14 in MAPK signaling. It appears that the molecular mechanisms of this disease rely on impairment of the function and cellular localization of lysosome-like organelles, again underscoring the importance of endosomal MAPK signaling in the biogenesis of lysosome-related organelles. However, not only in humans, but also in mice, an efficient MAPK signal transduction through the p14/MP1 complex appears to be important in the regulation of tissue homeostasis. Since a constitutive genetic ablation of p14 is embryonically lethal, conditional knockout mice lacking the expression of p14 in the epidermis were created. The live born mice die shortly after birth due to severe skin defects and rapid dehydration, reflecting the compromised terminal differentiation of the epidermis [157]. However, recent findings of the Sabatini group have revealed an unexpected role of the MP1/p14/p18 complex in signaling through the mammalian target of the Rapamycin (mTOR) pathway [158]. It was shown that this complex targets mTOR to lysosomes and is a prerequisite for amino acid-induced mTOR signaling. In light of these findings, one should thus be cautious when interpreting the results of gene knockdown or knockout studies of MP1/p14/p18 complex proteins, as the observed effect may well be due to impairment of both MAPK and mTOR signaling pathways.

### 2.4. β-Arrestins

The multifunctional adaptor proteins, ß-arrestins, were originally discovered as proteins that desensitize or terminate G protein coupled receptor (GPCR) signaling. Upon ligand stimulation, GPCRs are phosphorylated by the family of GPCR kinases (GRKs), and this event promotes the recruitment of ß-arrestins to the GPCRs. Consequently, the interaction with ß-arrestins sterically prevents a further coupling of GPRCs with the trimeric G proteins and disrupts the normal activation of the second messenger signaling cascade [159,160]. Later on, several other signaling pathways, e.g., Hedgehog, Wingless, Notch and TGFß pathways, were found to exploit the scaffolding functions of ß-arrestins (reviewed in [161]).

Four members of the ß-arrestin family have been described so far, and they show different, but overlapping, expression patterns. Arrestin 2 (ß-arrestin 1) and arrestin 3 (ß-arrestin 2) are ubiquitously expressed, whereas arrestin 1 (visual ß-arrestin) is localized to retinal rods and cones and arrestin 4 (X-arrestin) is found only in retinal cones. All four members are highly similar, sharing 70% sequence identity, and they are highly conserved across species (reviewed in [162]).

In addition to their role as desensitizers of GPCR signaling, ß-arrestins have been shown to mediate the clathrin-dependent endocytosis of seven transmembrane spanning receptors and to act as adaptors for different E3 ubiquitin ligases [163–166]. One of these is the E3 ligase, Mdm2, which catalyzes the ubiquitination of the ß2 adrenergic receptor (ß2AR) and of ß-arrestin 2 itself. Ubiquitination of ß2AR was found to be important for its degradation. Furthermore, ubiquitination of ß-arrestin 2 is indispensable for receptor internalization, since ubiquitinated ß-arrestin 2 is able to recruit certain components of the endocytic machinery, such as clathrin and activating protein 2 (AP-2) [165,167]. Moreover, the ubiquitination status of ß-arrestin 2 governs its association to the ß2AR and determines the stability of the ß-arrestin/GPCR complex. Permanently ubiquitinated ß-arrestin 2 does not dissociate from ß2AR, but internalizes with it to endosomes, whereas binding of the ß-arrestin 2 to the receptor is significantly increased after inhibition of deubiquitinating enzymes [164].

Following ß2 adrenergic receptor activation, the MAPK signaling cascade is activated in a biphasic way. The first phase of the activation is rapid and transient, with the signal peaking 2–5 min after stimulation. The second sustained phase is slower, lasting up to 30 min. The signal peaks 5–10 min after stimulation, and this phase of MAPK activation was found to be mediated by ß-arrestins [168,169]. ß-arrestins can scaffold all three kinases (C-Raf, MEK1 and ERK1/2) of the MAPK signaling cascade, facilitating the phosphorylation and activation of ERK1/2 and, at the same time, retaining ERK1/2 in the cytosol and prolonging the signaling events [152,170,171]. On the other hand, ß-arrestin is subjected to a negative feedback mechanism, during which active ERK phosphorylates its crucial Ser412 residue [172]. Cytosolic ß-arrestin is constitutively phosphorylated at this residue. Rapid dephosphorylation of Ser412 takes place upon agonist stimulation when ß-arrestin is recruited to the plasma membrane. Although it was found not to be required for receptor binding and receptor desensitization, dephosphorylation of Ser412 was shown to be essential for clathrin binding and receptor internalization [173]. Apart from ERK, ß-arrestin 2 was found to facilitate the activation of another MAPK, JNK3, by acting as a scaffold that brings together angiotensin II type 1A (AT1A) receptor, JNK3 and its upstream kinases, ASK1 and mitogen-activated protein kinase kinase 4 (MKK4), and clustering them together on endosomal structures after treatment with angiotensin II. Similarly to ERK, phosphorylated, active JNK3 then accumulates in the cytosol [174].

Having in mind all the aforementioned roles that ß-arrestins play in different signaling pathways, one would expect significant developmental defects after genetic ablation of the arrestins. However, ß-arrestin 1 knockout mice develop normally, but display increased cardiac contractility in response to adrenergic receptor agonists [175]. Similarly, ß-arrestin 2 knockout mice are viable and develop normally. Nevertheless, after administration of morphine, a prolonged analgesic effect in the knockout mice was observed. This was shown to be due to misregulated internalization and desensitization of the μ opioid receptor [176]. Although the single knockout mice models for ß-arrestin 1 and 2 appear healthy and normal, unless challenged, one can assume that ß-arrestins are functionally not completely interchangeable [161,177]. However, they can functionally compensate for each other in the developing mouse embryo to allow a normal development.

### 2.5. Fibroblast Growth Factor Receptor Substrate 2

FRS2 owes its name to the major and the first described function of this protein as a substrate for the fibroblast growth factor (FGF) receptor [178], although it has later been shown to be an important docking protein for many different RTKs [178–183]. FRS2 regulates downstream signaling by forming molecular complexes with other adaptor proteins and tyrosine phosphatases, and it seems to be a critical mediator of sustained ERK activity [184]. Its molecular structure is identical to the homologous protein, FRS3. On their N-terminus, both FRS proteins contain a putative myristoylation site, which is important for their membrane localization [178]. This sequence is followed by a phosphotyrosine binding domain, an anchor for binding to specific peptides of certain receptor tyrosine kinases with or without tyrosine phosphorylated residues [185]. For example, the binding to neurotrophin receptors TrkA and TrkB is dependent on the activation of the specific receptor and mediated by the tyrosine phosphorylated peptides [185–187]. In contrast, FRS binding to the FGF receptor is constitutive and involves unphosphorylated amino acids at the juxtamembrane domain of the FGF receptor [185,188]. The C-terminus of FRS proteins contains multiple tyrosine phosphorylation sites (six Tyr residues in FRS2 and five Tyr residues in FRS3). These residues, when phosphorylated by specific RTKs, recruit SH2-domain containing proteins, such as the adaptor protein, Grb2, and protein tyrosine phosphatase Shp2 [178,189]. The recruitment of Grb2 will eventually result in a strong activation of phosphatidylinositide 3 (PI3) kinase signaling pathway and a moderate activation of ERK signaling [189,190], whereas the recruitment of Shp2 causes a strong activation of the ERK signaling pathway [189]. Furthermore, FRS2 does not only function as a platform for the recruitment of proteins responsible for signal activation, but also for those involved in signal attenuation. Its scaffolding function enables Cbl, an ubiquitin ligase for RTKs, to come in close proximity of the FGF receptor. Cbl then ubiquitinates both the FGF receptor and FRS2 and directs them to degradation [191]. Therefore, by playing a dual role in both signal stimulation and attenuation, FRS2 is an important factor in assisting the FGF receptor in the regulation of the developmental processes. In agreement with this, FRS2 is ubiquitously expressed, with the highest expression level in brain, lung, kidney, ovary and testis and can be detected at virtually every developmental stage of a mouse [192]. In line with its important function during development, FRS2 knockout mice display embryonic lethality due to severe problems in gastrulation [180,193].

### 2.6. MAP Kinase Organizer 1

Whereas FRS2 plays a role in the most upstream part of the MAPK pathway by recruiting signaling components to the RTKs, MORG1 is a scaffold protein in the downstream part of the ERK cascade [194]. Interestingly, MORG1 was first isolated as a binding partner of MP1 [194], which is a very small scaffold protein without the capacity to independently facilitate the assembly of all the kinases in the ERK pathway [85]. Although MORG1 is composed of only 315 amino acids and exhibits a molecular mass of approximately 35 kDa, it is composed of as many as seven WD-40 domains, which form beta-propeller structures and serve as docking sites for many MAPK signal transducers. In this respect, MORG1 associates with ERK1/2, MEK1/2, C-Raf and B-Raf and accelerates downstream signaling from G protein-associated receptors in response to lysophosphatidic acid. Furthermore, it facilitates ERK1/2 activation when cells are stimulated with phorbol 12-myristate 13-acetate (PMA) or serum. However, MORG1 is dispensable for the downstream signaling of major tyrosine kinase receptors, such as the epidermal growth factor (EGF) and platelet-derived growth factor (PDGF) receptors [194].

MORG1 was also shown to be important for the regulation of the stability of hypoxia-inducible factor-1 (HIF-1) [195]. As a part of a transcriptional complex, HIF-1 activates the transcription of genes involved in the cellular adaptation to low oxygen availability. HIF-1 is composed of two subunits, the oxygen sensitive alpha- and beta-subunits. Under normal oxygen levels, the alpha subunit is hydroxylated by a family of prolyl hydroxylases (PHD). This creates a signal for ubiquitin ligases to exert ubiquitination that will finally target the protein for proteasomal degradation. MORG1 was found to bind to PHD3, and this interaction was shown to activate or stabilize PHD3. In this way, MORG1 assists PHD3 in the regulation of the protein levels of HIF-1α [195].

MORG1 is ubiquitously expressed, with the highest expression levels in heart, brain, liver, kidney and testis, while lower amounts were detected in lung, spleen and skeletal muscle [194]. MORG1 knockout mice display an embryonically lethal phenotype, due to severe neuronal developmental defects, whereas heterozygous MORG1^+/−^ mice are normal [196–198]. However, when compared to the wild-type littermates, heterozygous MORG1^+/−^ mice are partially protected from renal and cerebral ischemia-reperfusion injury [197,198]. Furthermore, expression of MORG1 was found to be reduced in human brain tissue with ischemic damage, while astrocytes in the surrounding brain tissue showed a strong MORG1 expression [196]. Decreased MORG1 expression might be an intrinsic mechanism of the injured tissue to activate the genetic program, which would enable brain recovery with the help of the increased HIF-1alpha expression and adaptation to hypoxia. These observations put MORG1 into the class of putative therapeutic targets with the aim to limit renal or cerebral injury after ischemia-reperfusion.

### 2.7. Flotillin-1, a Novel MAPK Scaffolding Protein

Membrane microdomains, also known as lipid rafts, participate in cellular signaling events by providing a specialized surrounding where membrane receptors, their respective signaling partners and adaptor proteins meet to initiate downstream signaling. Due to their enrichment in sphingolipids and cholesterol, these microdomains exhibit a so-called liquid ordered state in the membranes and are able to float in low density fractions of detergent resistant membrane (DRM) preparations [199,200]. Proteins of the flotillin family (flotillin-1 and −2) constitutively associate with membrane microdomains [201,202] and are therefore frequently used as marker proteins for those microdomains. For our recent review on flotillin function, please see [203].

Structurally, flotillins are organized in two major domains. The *N*-terminal globular SPFH (stomatin/prohibitin/flotillin/HflK/C), also called PHB (prohibitin homology), domain contains acylated residues and hydrophobic stretches that, together with the *C*-terminal flotillin-domain, enable flotillins to firmly associate with membrane microdomains. In addition, the predicted coiled-coil structures in the *C*-terminus of flotillins are essential for proper homo- and hetero-oligomerization [204–210]. Subcellularly, flotillins localize to the plasma membrane, vesicular/endosomal structures and to the nucleus [209–215]. However, the subcellular localization of flotillins is rather dynamic and depends on the cell type and external stimuli.

Interestingly, flotillins are downstream transcriptional targets of ERK signaling. Their transcription is regulated not only by ERK, but also by the retinoic X receptor complexes, which is detectable as an enhanced activity of the flotillin promoter [216]. On the other hand, flotillins, especially flotillin-1, are important regulators of ERK signaling (see below). It is noteworthy that, upon transient depletion of flotillin-1 in HeLa cells, the expression of cyclin D1 upon growth factor treatment is impaired [217,218]. This correlates well with the observation of Lin *et al.*, showing that a knockdown of flotillin-1 in breast cancer cells yields a reduced expression of cyclin D1 and results in an arrest of the cell cycle in the G1/S phase [218].

Flotillins have been functionally implicated to participate in several cellular processes, such as clathrin-independent endocytosis [212,219], organization of the actin cytoskeleton [208,209,220], cellular adhesion [209] and various signaling processes [209,217,221–224]. Stimulation of cells with growth factors, such as EGF, results in Src family kinase-mediated phosphorylation of flotillins and their translocation from the plasma membrane to late endosomes [204,225]. This process is dependent on the proper hetero-oligomerization of flotillins and the phosphorylation of Tyr163 in flotillin-2 and Tyr160 in flotillin-1, and a point mutation of Tyr163 into Phe results in constitutive localization of flotillin-2 to the plasma membrane [204,209].

Recently, our group and others found implications of flotillins in MAPK signaling pathways, for example, in EGF receptor [217], insulin receptor [226], TrkA receptor [223] or IgE receptor signaling [222]. Sugawara and colleagues investigated the GPCR-mediated p38/MAPK signaling pathway and showed that flotillins are Gαq binding proteins that positively modulate G_q_ signaling [224]. Furthermore, in the context of fibroblast growth factor receptor (FGFR)-mediated MAPK downstream signaling, flotillin-1 interacts with both members of the FRS family of scaffold proteins (FRS2 and FRS3) [227]. Upon growth factor treatment of cells, FRS2 is phosphorylated and serves as an adapter protein, which recruits several signaling proteins that induce Ras-mediated MAPK signaling. In addition, it has been suggested that ERK1/2 acts as a negative feedback regulator on FRS2 [193,228,229]. Upon flotillin-1 depletion, tyrosine phosphorylation of FRS2 is increased, implicating that the flotillin-1/FRS2 signaling complex is required for proper growth factor signaling [227].

**Figure 3:**
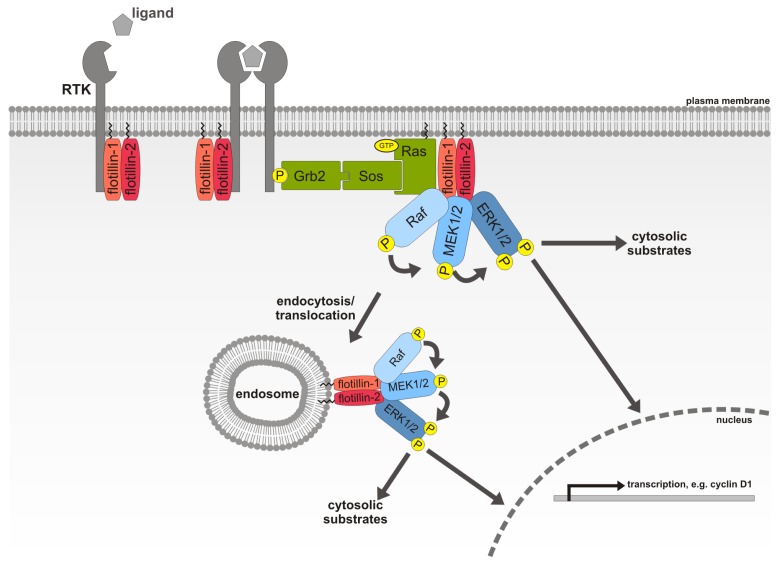
Flotillins are novel scaffolding proteins for the ERK/MAPK pathway. Both flotillins associate with the epidermal growth factor receptor (EGFR) and are necessary for a proper activation of the receptor and its downstream MAPK signaling pathway. Flotillin-1, which is found in hetero-oligomers with flotillin-2, is a scaffolding protein for C-Raf, MAPK/ERK kinase 1 (MEK1) and extracellular signal-regulated kinase 2 (ERK2) independently of kinase suppressor of Ras (KSR1). A nuclear target gene of the flotillin scaffolded MAPK cascade is cyclin D1, but flotillins can also influence cytosolic ERK activity.

Recently, we described a dual role for flotillin-1 during EGFR activation and MAPK downstream signaling [217]. Early, after growth factor stimulation, transient loss of flotillin-1 results in a diminished activation of the receptor tyrosine kinases, as well as impaired ligand-induced receptor clustering prior to its internalization, which indicates an important role for flotillin-1 during receptor tyrosine kinase activation. Later on, flotillin-1 binds the core components of the MAPK cascade C-Raf, MEK1 and ERK2 simultaneously and independently of KSR1 (Figure 3). However, it is still unclear how exactly flotillin-1 associates with the said MAPK components and whether this association changes upon growth factor treatment. Nevertheless, the ability of flotillin-1 to bind the three tiers of the MAPK pathway simultaneously, modulate ERK activation and regulate transcriptional regulation downstream of ERK defines flotillin-1 as a MAPK scaffolding protein [216,217].

## 3. MAP Kinase Scaffolders: What Are They Good for?

Originally, a simplified view of signaling pathways with a linear flow and amplification was presented, which can still be found in many text books, *etc*. However, recent research efforts have revealed that rather than utilizing linear cascades, signaling proceeds through intertwining communication networks. The plethora of scaffolding proteins that have been identified in recent years are required for many aspects of signaling. Scaffolders not only increase the efficiency and specificity of signaling, but they also facilitate the crosstalk between different signaling pathways. For example, the MP1/p14 complex plays an important role in the crosstalk between Rac/p21-activated kinase 1 (PAK1) and ERK signaling pathways. It regulates the PAK1-dependent activation of MEK1 in response to adhesion, thus representing a point of convergence for integrating growth factor signaling and cell adhesion to extracellular matrix (ECM) [230,231]. Importantly, the kinetics (amplitude, duration, *etc*.) of a signaling response is extremely important in regulating the final cellular outcome of the signaling, as, e.g., MAPK activation can result in either proliferation or differentiation of the target cells.

But why do we need so many different scaffolding proteins for a single signaling pathway, such as the MAPK cascade? These scaffolds are usually part of and actually even vital for the formation of multiprotein signaling complexes, and they frequently even bind to the same cascade components. How can a specific function still be accomplished? Upon a closer look, the different scaffolds show many features that distinguish them from the others. Some scaffolds show a very specific cellular localization and may thus regulate or restrict the signaling in specific subcompartments, as, for example, the MP1/p14 scaffold in endosomes, Sef1 in the Golgi or GIT1 (G protein coupled receptor kinase interacting 1), RACK1 (receptor for activated protein C kinase 1) and paxillin in focal adhesions [232]. Furthermore, some scaffolds are constitutive, whereas others are stimulus-dependent. Even within one scaffold, different components may bind either constitutively or only after an extracellular stimulus. It may even be that some scaffolds only respond to specific stimuli, but not to others, as has been shown for MORG1 [194]. The scaffolds also differ in the number of signaling proteins they are able to bind. For example, the MP1/p14 scaffold is a two-component scaffolder that binds MEK1 and ERK1, whereas KSR1 and flotillin-1 can bind at least three signaling tiers. In the case of MP1, its specificity towards MEK1/ERK1 may facilitate specific cellular responses by means of activation of ERK1, but not ERK2 in endosomes.

Some scaffolders, such as MORG1 and flotillin-1, appear even to be “superscaffolds” that are not only capable of binding to the MAPK components, but also to other scaffolds. For example, MORG1 binds to Raf, MEK and ERK, but also associates with the MP1 scaffold in the endosomes [194], whereas RACK1 can bind Raf, MEK and ERK, but it also associates with MP1 [232]. Similarly, flotillin-1 directly associates with Raf, MEK and ERK, but it is also found in a complex with KSR1 [217]. Intriguingly, flotillin-1 also interacts with both FRS2 that has been implicated as a MAPK scaffold and with MORG1 (our unpublished data). However, it has not been studied so far if flotillin-1 is also capable of interacting with MP1, but its interaction with MORG1 may point to at least a functional, if not direct, interaction. Due to the fact that the localization of flotillin-1 changes during signaling in that it becomes endocytosed from the plasma membrane into endosomes upon growth factor stimulation, flotillin-1 may be capable of functioning as a mediator between different scaffolds that show a more specific and signal-independent localization in a certain cellular compartment. The molecular details of this potential flotillin-1 function still need to be clarified in detail, but it is plausible that flotillin-1 acts as a master scaffolder that plays an important role in the regulation of other MAPK scaffolds.
